# Scalable Preparation of Enantioenriched (*S*)-5-methylhept-2-en-4-one. Synthesis and Aroma Properties of Achiral Analogues Thereof

**DOI:** 10.3390/molecules24244497

**Published:** 2019-12-08

**Authors:** Eva Puchľová, Michal Dendys, Ivan Špánik, Peter Szolcsányi

**Affiliations:** 1 Department of Organic Chemistry, Slovak University of Technology, Radlinského 9, SK-812 37 Bratislava, Slovakia; puchlova.eva@gmail.com (E.P.); michaldendys@gmail.com (M.D.); 2 Department of Analytical Chemistry, Slovak University of Technology, Radlinského 9, SK-812 37 Bratislava, Slovakia; ivan.spanik@stuba.sk

**Keywords:** hazelnut, enzymatic hydrolysis, fragrant enones, allylic oxidation, sensory analysis

## Abstract

(*S*)-5-Methylhept-2-en-4-one is a key flavour compound in hazelnuts. We have performed its chiral-pool-based chemoenzymatic synthesis with 39% overall yield (73% *ee*). The four-step aldol-based sequence avoids the use of highly reactive and/or toxic reagents, does not require anhydrous conditions and uses only distillation as the purification method. Thus, such methodology represents a green and scalable alternative to only two stereoselective approaches towards this natural product known so far. In addition, we have designed and prepared a set of new (di)enones as achiral synthetic analogues of the title compound. The results of their sensory analyses clearly show that relatively minor structural changes of the natural molecule significantly alter its olfactory properties. Thus, simple (poly)methylation completely changes the original hazelnut aroma of (*S*)-5-methylhept-2-en-4-one and shifts the odour of its analogues to eucalyptus, menthol, camphor, and sweet aroma.

## 1. Introduction

**Scheme 1:**
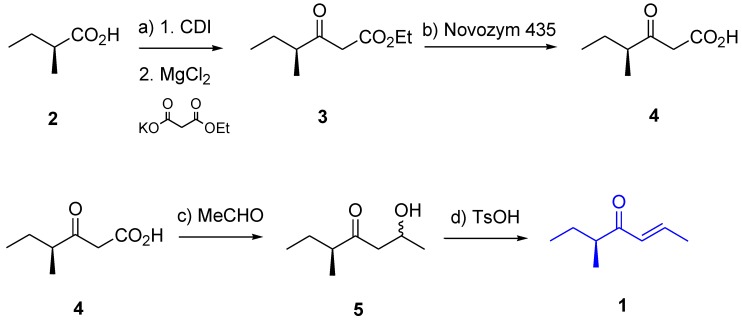
Synthesis of enantioenriched (*S*)-5-methylhept-2-en-4-one **1**. Reagents and conditions: (**a**) CDI (1.2 equiv), THF, RT, 4 h; potassium ethyl malonate (1.5 equiv), MgCl_2_ (1.5 equiv), THF, 60 °C, 5 h; then 50 °C, 5 h → RT, overnight, vacuum distillation, 90% (2 steps); (**b**) Novozym 435 (5% wt), phosphate buffer, RT, 22 h, crude (100% GC, 88% *ee*); (**c**) MeCHO (1.1 equiv), TBAHSO_4_ (0.005 equiv), RT → 40 °C, 21 h, vacuum distillation, 53%; and (**d**) *p*-TsOH·H_2_O (0.05 equiv), cyclohexane, 70 °C, 2.5 h, vacuum distillation, 82% (73% *ee*).

(*E*)-5-Methylhept-2-en-4-one is a key flavour compound in fruits of hazelnut trees *Corylus maxima* and *C. avellana*. The enantiomeric content in hazelnuts of different geographic origin ranges from 54 to 73% *ee* in favour of (*S*)-enantiomer **1** (Scheme 1). Due to unique sensory properties, it has found practical applications in perfumery, flavour and food industry. Moreover, it is recently used for analytical purposes as a key marker for the detection of adulteration of olive oil and/or authenticity evaluation of hazelnut-based products [1]. Consequently, there is an emerging interest in its straightforward and cost-efficient stereoselective synthesis as a reliable source of an enantioenriched **1** for direct use.

To date, only three enantioselective syntheses of (*S*)-5-methylhept-2-en-4-one **1** are known [2,3,4], and all employ enantiomerically pure (*S*)-2-methylbutanol as substrate, with 40% overall yield (75% and 92% *ee*). However, these strategies rely on synthetic methods either requiring strictly anhydrous conditions (organolithium addition, hydride reduction) or use/generate toxic reagents/waste (chromium salts), and thus, severely limiting their scaling potential. Therefore, green yet efficient approaches towards the synthesis of enantioenriched **1** are clearly needed.

Moreover, its relatively simple structure associated with unique flavour profile is an ideal scaffold for the design of novel analogues. Advantageously in this context, the chirality of **1** has a negligible influence on the quality of its sensory properties as the odours of all stereoisomers are typical of hazelnuts [5]. Thus, rationally designed, preferably achiral derivatives of **1** could provide a useful insight into the structure–sensory correlations (SAR) necessary for future identification of the key molecular features associated with the hazelnut aroma.

## 2. Results and Discussion

### 2.1. Chemoenzymatic Preparation of Enantioenriched **1**

Inspired by the aldol-based approach [6] to racemic 5-methylhept-2-en-4-one [7,8,9,10,11,12,13,14,15,16], we have designed our synthetic strategy towards enantioenriched **1** with key focus on the suppression of the associated epimerisation and use of naturally occurring substrates throughout the multigram sequence (Scheme 1). Thus, the initial activation [17] of natural (*S*)-2-methylbutanoic acid **2** (88% *ee*) followed by one-pot condensation of intermediary acyl imidazole with potassium ethyl malonate furnished the β-ketoester **3** in 90% yield in 2 steps. While the extensive screening of various Lewis/Brønsted acids or bases for hydrolysis of **3** yielded completely racemic product only, its enzymatic hydrolysis [18] with immobilised lipase from *Candida antarctica* afforded crude β-ketoacid **4** with preserved stereointegrity and quantitative yield (100% GC, 88% *ee*). This intermediate was directly used in the decarboxylative Knoevenagel condensation with acetaldehyde to furnish ketol **5** in 53% yield after distillation. The extensive screening of final acid-catalysed dehydration [19] of the latter afforded the enantioenriched **1** in 82% yield with an acceptable epimerisation (73% *ee*) when using *p*-toluene sulfonic acid in hydrocarbon solvent (see Supplementary Material). Overall, the total yield of (*S*)-5-methylhept-2-en-4-one **1** obtained in four steps reached 39%, with the enantiomeric purity conforming to the purest material isolated from natural sources (*vide supra*). Moreover, our protocol is based on generally innocuous synthetic transformations, avoids the use of highly reactive organometallic and/or toxic reagents, does not require strictly anhydrous reaction conditions and uses vacuum distillation as the only purification method. Thus, such methodology represents a green and scalable alternative to the very few approaches towards the stereoselective synthesis of enantioenriched **1** known so far.

### 2.2. Synthesis of Achiral Analogues **6**–**9**

**Figure 1:**
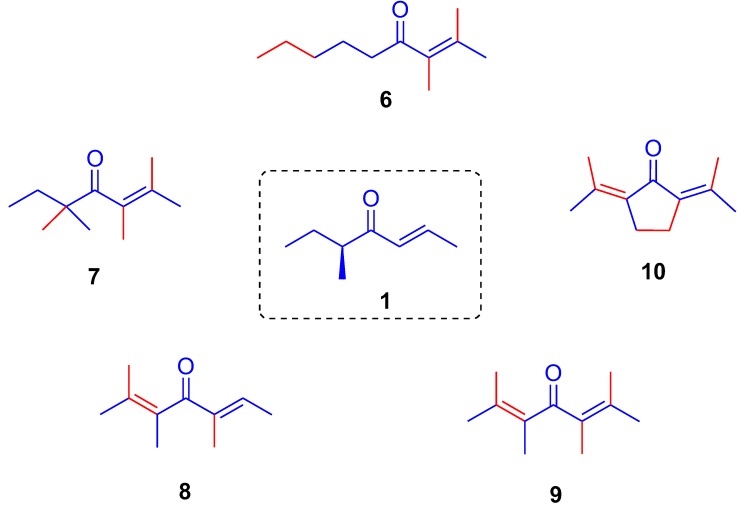
Achiral analogues **6**–**10** of (*S*)-5-methylhept-2-en-4-one **1**.

In the next part, we were further interested in the design, synthesis and properties of new achiral analogues of **1**. Our aim was to gain an insight into the structure–sensory correlations (SAR) necessary for identifying the key molecular features associated with its unique hazelnut aroma. Thus, we have designed a small set of (poly)alkylated enones **6**–**9**, all featuring the structural core of **1** with extra added (m)ethyl groups. These included previously unknown acyclic enones **6**, **7** and dienones **8**, **9** as well as the known [20,21,22] cyclic dienone **10** for comparison purposes (Figure 1).

**Scheme 2:**
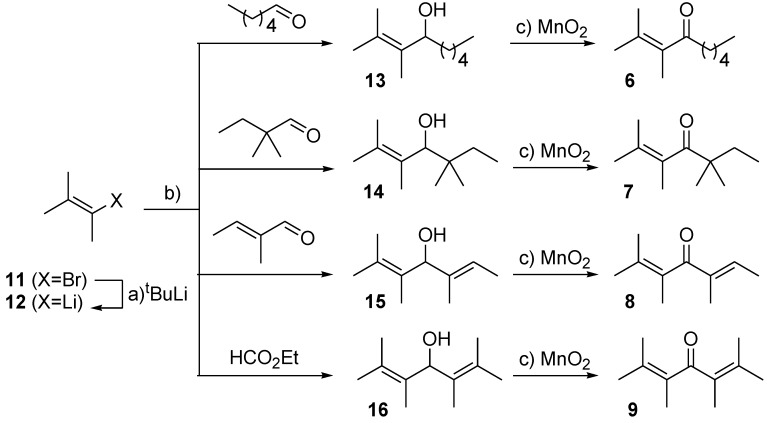
Preparation of new achiral analogues **6**–**9**. Reagents and conditions: (**a**) ^t^BuLi (2.1 equiv), THF, −78 °C, 30 min → RT, 1 h, crude; (**b**) **12** (1.05 equiv), THF, −50 or −30 °C → RT, 15 h, vacuum distillation, **13** (84%), **14** (67%), **15** (84%), **16** (71%); and (**c**) MnO_2_ (20 equiv), pentane, RT, 48 h to 4 d, crude, **6** (71%), **7** (74%), **8** (74%), **9** (76%).

At this preliminary stage, we chose the most direct synthetic route employing reliable and powerful (organo)metallic transformations regardless of their non-greenness. Thus, our preparation of new (di)enones **6**–**9** is based on a short two-step protocol: in the first step, the addition of 1,2-dimethylpropenyllithium **12**, freshly made [23] by halogen-metal exchange [24] of commercial 2-bromo-2-methylbutene **11** and *tert*-butyl lithium, to a range of commercial carbonyl electrophiles furnished corresponding (di)allyl alcohols **13**–**16** in good to high yields (67–84%). These were finally transformed by allylic oxidation with manganese dioxide to target (di)enones **6**–**9** in overall yields of 50–62% over two steps (Scheme 2, see Supplementary Material).

### 2.3. Olfactory Evaluation

With new (di)enones **6**–**9** in hands, we have undertaken their sensory evaluation. For comparison purposes, we have also included (di)allyl alcohols **13**–**16** and known cyclic dienone **10** in the olfactory screening. The results are summarised in Table 1. Firstly, and most importantly, methylation/alkylation of target molecule led to a complete change of olfactory properties as none of its analogues **6**–**9** exhibited any hazelnut aroma. Thus, their sensory characteristics can be divided into three distinct classes according to major odour. (Di)enones **7**–**10** possess a fresh camphoraceous–minthy–eucalypty scent with additional floral, sweet and spicy tones. Interestingly, similar major aspects were also exhibited by allyl alcohol **14** with accompanying terpenic notes. While major sweet aroma with tropical fruit notes is typical for enone **6**, an analogous alcohol **13**, however, exhibits an earthy scent of root vegetables with an interesting tone of dried poppy heads. Into the latter odour group also belong diallyl alcohols **15** with celery aspect and **16** with potato note, respectively. The obtained results clearly show the shift of the hazelnut aroma of **1** to the potentially exploitable eucalyptus, camphor, menthol, and/or sweet odours exhibited by volatile new (di)enones **6**–**9**. In addition, the analogous (di)allyl alcohols **13**, **15**–**16** also display potentially useful earthy aromas strongly reminiscent of root vegetables.

## 3. Materials and Methods

### 3.1. General Experimental

Chemicals and reagents were purchased from commercial sources (Alfa Aesar, Sigma-Aldrich) and were used without further purification. In case of anhydrous solvents, these were prepared either by filtration through a column of activated alumina or by standing over activated 4Å molecular sieves and stored under argon atmosphere. Hexanes refer to a mixture of C-6 alkanes (b.p. 60–80 °C). Yields refer to chromatographically and spectroscopically (^1^H NMR) homogeneous material, unless otherwise stated. Reactions were monitored by thin layer chromatography (TLC) carried out on aluminium sheets pre-coated with silica gel 60 F_254_ (Merck) or aluminium oxide 60 F_254_ (neutral, Merck). Visualisation was performed using shortwave UV light followed by dipping TLC plates in either basic solution of KMnO_4_, acidic solution of vanillin or acidic solution of ceric ammonium nitrate followed by heating with a heat gun. Flash column chromatography (FLC) was performed using Silica Gel 60 (particle size 0.040–0.063 mm). NMR spectra were recorded in CDCl_3_ on a Varian INOVA 300 (300 MHz for ^1^H, 75 MHz for ^13^C nuclei) or Varian VNMRS 600 (600 MHz for ^1^H, 151 MHz for ^13^C nuclei) NMR spectrometer and were correctly shifted using residual non-deuterated solvent or tetramethylsilane as an internal reference (CHCl_3_: δ_H_ = 7.26 ppm, δ_C_ = 77.16 ppm (central peak of a 1:1:1 triplet), TMS: δ_H_ = δ_C_ = 0.00 ppm). Chemical shifts (δ) are quoted in ppm. LC-MS analyses were performed on Agilent 1200 Series instrument equipped with a multimode MS detector using the MM ESI/APCI ionisation method (column Zorbax Eclipse XDB-18, 150 × 4.6 mm, particle size 5 μm, eluent water with 0.1% HCO_2_H/CH_3_CN, 70:30, flow 1.5 mL/min). GC analyses were performed on a gas chromatograph Agilent 7820A equipped with FID and a split–splitless injector (column DB-5 30 m × 0.25 mm × 0.25 µm, injection 0.1 µL, split 20:1, temperature gradient 40 °C (2 min) → 15 °C/min → 220 °C (15 min), carrier gas H_2_, flow 1.2 mL/min). Chiral GC analyses were performed on a gas chromatograph Agilent 7890A equipped with FID and a split–splitless injector (column Cyclosil-B 30 m × 0.32 mm × 0.25 µm, injection 0.2 µL, split 50:1, temperature gradient 40 °C (0 min) → 10 °C/min → 80 °C (0 min) → 25 °C/min → 220 °C (2 min), carrier gas H_2_, flow 2.0 mL/min). GC-MS analyses were performed on a gas chromatograph Agilent 7890A and coupled with Agilent 5975C inert MSD with Triple-Axis Detector (column DB-Wax 30 m × 0.25 mm × 0.15 µm, injection 1 µL, split 20:1, temperature gradient 40 °C (2 min) → 15 °C/min → 220 °C (15 min), carrier gas H_2_, flow 1.2 mL/min). High-resolution mass spectra (HR-MS) were recorded on a Thermo Scientific Orbitrap Velos mass spectrometer with a heated electrospray ionisation (HESI) source in positive and/or negative mode. FTIR spectra were obtained on a Nicolet 5700 spectrometer (Thermo Electron) equipped with a Smart Orbit (diamond crystal ATR) accessory using the reflectance technique (4000–400 cm^–1^). The sensory analysis was performed by authors in a clean and odourless environment at 22 °C. The prepared compounds were evaluated as 10% solutions in aqueous ethanol (95% *w*/*w*) by using testing strips.

### 3.2. Synthetic Procedures and Analytical Data

(*S*)-Ethyl 4-methyl-3-oxohexanoate (**3**) A mixture of potassium ethyl malonate (100.0 g, 0.587 mol, 1.5 equiv) was treated with magnesium chloride (56.0 g, 0.587 mol, 1.5 equiv) in tetrahydrofuran (360 mL), and the resulting grey slurry was stirred at 60 °C for 5 h. During that time, in a separate reaction vessel, a solution of (*S*)-2-methylbutanoic acid **2** (88% *ee*, 40.0 g, 0.343 mol) in THF (160 mL) was added to a solution of carbonyldiimidazole (66.0 g, 0.407 mol, 1.2 equiv) in THF (140 mL) and the resulting yellow solution was stirred at 28 °C for 4 h. Then, after 5 h reaction time, a THF solution containing a mixture of malonate and MgCl_2_ was cooled to RT and a THF solution of crude acyl imidazole formed from acid **2** was added dropwise over 30 min. The resulting white suspension was stirred at 50 °C for 5 h and subsequently at RT overnight. The reaction mixture was then added to 1M aqueous HCl solution (1600 mL). The resulting pale-yellow solution was stirred at RT for 30 min, ethyl acetate (600 mL) was added, phases were separated, and aq ueous layer was extracted with EtOAc (600 mL). Combined organic extracts were sequentially washed with 1M aq ueous HCl solution (400 mL), water (500 mL), 2% aqueous Na_2_CO_3_ solution (700 mL), water (500 mL) and brine (500 mL), subsequently dried over anhydrous Na_2_SO_4_ and concentrated in vacuo to give pale-yellow oil (76.23 g). The crude product was purified by vacuum distillation (b.p. 64–67 °C/3.4 mbar) to yield (*S*)-ketoester **3** (60.54 g, 90%) as a colourless liquid; δ_H_ (300 MHz, CDCl_3_) 4.19 (q, *J* = 7.1 Hz, 2H, OCH_2_CH_3_), 3.47 (s, 2H, H-2), 2.58 (m, *J* = 6.8 Hz, 1H, H-4), 1.79–1.62 (m, 1H, H-5A), 1.42 (m, 1H, H-5B), 1.27 (t, *J* = 7.1 Hz, 3H, OCH_2_CH_3_), 1.10 (d, *J* = 6.9 Hz, 3H, Me), 0.90 (t, *J* = 7.4 Hz, 3H, H-6); δ_C_ (75 MHz, CDCl_3_) 206.6 (C-3), 167.4 (C-1), 61.3 (OCH_2_), 48.1 (C-4), 47.8 (C-2), 25.7 (C-5), 15.5 (Me), 14.2 (OCH_2_*C*H_3_), 11.5 (C-6), NMR spectra of (*S*)-**3** are in full accordance with literature data [25,26,27] for racemic **3**; in addition, signals of the enol form of **3** are clearly detectable in both proton and carbon spectra measured in deuterochloroform: δ_H_ (300 MHz, CDCl_3_) 12.11 (d, *J* = 0.7 Hz, 1H, OH), 4.97 (s, 1H, H-2), 4.18 (q, *J* = 7.1 Hz, 2H, OCH_2_CH_3_), 2.15 (m, *J* = 7.0 Hz, 1H, H-4), 1.79–1.62 (m, 1H, H-5A), 1.42 (m, 1H, H-5B), 1.29 (t, *J* = 7.1 Hz, 3H, OCH_2_CH_3_), 1.12 (d, *J* = 6.9 Hz, 3H, Me), 0.89 (t, *J* = 7.4 Hz, 3H, H-6); δ_C_ (75 MHz, CDCl_3_) 182.7 (C-3), 173.0 (C-1), 88.1 (C-2), 60.0 (OCH_2_), 41.2 (C-4), 27.1 (C-5), 17.7 (Me), 14.4 (OCH_2_CH_3_), 11.7 (C-6); GC: t_R_ = 7.73 min (keto-form), t_R_ = 7.54 min (enol-form); GC-MS: *m/z* (%) 172 (6, M^+^), 157 (1), 144 (9), 127 (3), 115 (27), 98 (5), 85 (31), 69 (12), 57 (100), 43 (36).

(*S*)-4-Methyl-3-oxohexanoic acid (**4**) To a solution of ketoester **3** (8.0 g, 46.5 mmol) in an aqueous sodium phosphate buffer (32 mL, pH~7) was added Novozym 435 (400 mg) and the suspension was stirred at RT for 22 h, while the pH was kept neutral by addition of aqueous NaOH. Solids were filtered off and the filtrate containing ketoacid **4** (100% GC yield, 88% *ee*) was directly used for the subsequent Knoevenagel condensation with acetaldehyde. The enantiomeric purity of crude **4** was determined by chiral GC via (*S*)-3-methylpent-2-on (t_R_ = 3.53 min) formed in situ by thermal decarboxylation of **4** during analysis.

(*S*)-2-Hydroxy-5-methylhept-4-one (**5**) To a solution of crude ketoacid **4** (8 g, 46.5 mmol) in an aqueous phosphate buffer (42.5 mL) was added tetrabutylammonium hydrogen sulphate (79 mg) and the pH was adjusted to approx. 8 by aqueous NaOH solution. Acetaldehyde (2.6 mL, 51.4 mmol, 1.1 equiv) was added and the resulting soln. was stirred at RT for 1.5 h and then at 40 °C for 21 h. The reaction mixture was extracted with diethyl ether (3 × 70 mL), separated organic layer was dried over anhydrous Na_2_SO_4_ and the solvent was evaporated in vacuo to yield pale-yellow liquid (6.865 g). The crude product was purified by vacuum distillation (b.p. 63-64 °C/3.6 mbar) to afford ketol **5** (3.577 g, 53%) as a colourless liquid; δ_H_ (600 MHz, CDCl_3_) 4.23–4.17 (m, 1H, H-2), 3.01 (bs, 1H, exchange with D_2_O, OH), 2.62 (ddd, *J* = 17.8, 13.0, 2.8 Hz, 1H, H-3a), 2.51 (ddd, *J* = 17.8, 13.5, 9.0 Hz, 1H, H-3b), 2.46–2.39 (m, 1H, H-5), 1.72–1.63 (m, 1H, H-6a), 1.43–1.35 (m, 1H, H-6b), 1.18 (dd, *J* = 6.4, 0.7 Hz, 3H, H-1), 1.06 (dd, *J* = 7.0, 1.3 Hz, 3H, Me), 0.87 (t, *J* = 7.5 Hz, 3H, H-7), NMR spectrum is in accordance with the literature data [28]; GC: t_R_ = 7.67 min; GC-MS: *m/z* (%) 144 (2, M^+^), 116 (4), 103 (18), 87 (85), 85 (26), 69 (23), 57 (75), 43 (100).

(*E*,*S*)-5-Methylhept-2-en-4-one (**1**) To a mixture of ketol **5** (2.85 g, 19.8 mmol) in cyclohexane (49 mL) was added *p*-toluenesulfonic acid monohydrate, (190 mg, 1.0 mmol, 0.05 equiv) and resulting mixture was stirred at 70 °C for 2.5 h. Subsequently, the mixture was washed with saturated aqueous NaHCO_3_ solution (25 mL), aqueous phase was extracted with diethyl ether (3 × 60 mL) and organic phase was dried over anhydrous Na_2_SO_4_ and concentrated in vacuo to give a colourless liquid (2.60 g). The crude product was purified by bulb-to-bulb vacuum distillation (b.p. 75–76 °C/4 mbar) to afford a (*S*)-enantioenriched enone **1** (2.03 g, 82% yield, 73% *ee*) as a colourless liquid; (the (S)-configuration of **1** as the major enantiomer was determined by the comparison with the analytical sample of (S)-**1** prepared independently from (*S*)-2-methylbutanol by previously reported stereoselective synthesis, cf. Ref. [4]); δ_H_ (300 MHz, CDCl_3_) 6.85 (dq, *J* = 15.6, 6.9 Hz, 1H, H-2), 6.16 (dd, *J* = 15.6, 1.7 Hz, 1H, H-3), 2.62 (m, *J* = 6.8 Hz, 1H, H-5), 1.86 (dd, *J* = 6.9, 1.7 Hz, 3H, H-1), 1.74–1.59 (m, 1H, H-6A), 1.46–1.25 (m, 1H, H-6B), 1.04 (d, *J* = 6.9 Hz, 3H, Me), 0.84 (t, *J* = 7.4 Hz, 3H, H-7); δ_C_ (75 MHz, CDCl_3_) 203.9 (C-4), 142.3 (C-2), 130.7 (C-3), 45.4 (C-5), 26.2 (C-6), 18.3 (C-1), 16.2 (Me), 11.8 (C-7), NMR spectra of (*S*)-**1** are in full accordance with the literature data [2]; Chiral GC: t_R_ = 5.74 min for (*S*)-**1** (t_R_ = 5.59 min for (*R*)-**1**); GC-MS: *m/z* (%) 126 (1, M^+^), 111 (11), 98 (12), 69 (100), 57 (4), 41 (21).

### 3.3. General experiment for the preparation of (di)allyl alcohols (**13**–**16**)

To a freshly prepared solution of 1,2-dimethylpropenyl lithium **12** in anhydrous THF was added the respective aldehyde or ester dropwise at low temperature over 5 min. under argon. The mixture was gradually warmed to RT and stirred overnight. The reaction was quenched with saturated aqueous NH_4_Cl solution, diluted with water and extracted with diethyl ether. Combined organic extracts were dried over MgSO_4_ and concentrated in vacuo (34 °C, 650 → 250 mbar). Crude product was purified by bulb-to-bulb vacuum distillation to furnish a corresponding allyl alcohol **13**–**16**.

2,3-Dimethylnon-2-en-4-ol (**13**) **12** (1.35 mmol, 1.05 equiv), THF (2 mL), −50 °C, hexanal (0.16 mL, 1.28 mmol), RT, overnight, NH_4_Cl (5 mL), H_2_O (5 mL), Et_2_O (3 × 10 mL), vacuum distillation (130 °C, 50 mbar), alcohol **13** (165 mg, 84%) as colourless oil; R_f_ (hexanes/AcOEt 4:1) 0.43; ν_max_ (ATR) 3342 (OH), 2955, 2927, 2859, 1457, 1375, 1010 cm^−1^; δ_H_ (300 MHz, CDCl_3_) 4.63 (t, *J* = 6.96 Hz, 1H, H-4), 1.71, 1.67, 1.61 (s, 2 × m, 3 × 3H, H-1, 2 × Me), 1.40 (m, 8H, H-5, H-6, H-7, H-8), 0.89 (m, 3H, H-9); δ_C_ (75 MHz, CDCl_3_) 129.6, 127.4 (C-2, C-3), 71.2 (C-4), 35.1 (C-5), 31.9 (C-7), 25.6 (C-6), 22.7 (C-8), 21.1, 19.8 (C-1, Me); 14.1 (C-9), 11.6 (Me); *m/z* (ESI) 153 (100, M-OH^+^), 154 (14%); HR-MS (HESI): M^+^, found 170.1665. C_11_H_22_O requires 170.1665.

2,3,5,5-Tetramethylhept-2-en-4-ol (**14**) **12** (2.62 mmol, 1.05 equiv), THF (2 mL), −30 °C, 2,2-dimethylbutanal (0.31 mL, 2.5 mmol), RT, overnight, NH_4_Cl (10 mL), H_2_O (10 mL), Et_2_O (3 × 15 mL), vacuum distillation (130 °C, 80 mbar), alcohol **14** (285 mg, 67%) as yellowish oil; R_f_ (hexanes/AcOEt 4:1) 0.59; ν_max_ (ATR) 3398 (OH), 2962, 2917, 2879, 1462, 1373, 1002 cm^−1^; δ_H_ (300 MHz, CDCl_3_) 4.42 (d, *J* = 3.75 Hz, 1H, H-4), 1.69, 1.66 (2 × m, 3H, 6H, H-1, 2 × Me), 1.35 (d, *J* = 3.75 Hz, OH), 1.31 (m, 2H, H-6), 0.86 (m, 3H, H-7), 0.88, 0.80 (2 × s,2 × 3H, 2 × Me); δ_C_ (75 MHz, CDCl_3_) 128.4, 129.2 (C-2, C-3), 76.9 (C-4), 39.7 (C-5), 31.9 (C-6), 23.5, 22.6 (2 × Me), 21.4, 21.3 (C-1, Me); 14.6 (Me), 8.5 (C-7); *m/z* (ESI) 153 (100, M-OH^+^), 154 (12%); HR-MS (HESI): M^+^, found 170.1665. C_11_H_22_O requires 170.1665.

(*E*)-2,3,5-Trimethylhept-2,5-dien-4-ol (**15**) **12** (1.35 mmol, 1.05 equiv), THF (2 mL), −45 °C, (*E*)-2-methylbutanal (0.13 mL, 1.28 mmol), RT, overnight, NH_4_Cl (5 mL), H_2_O (5 mL), Et_2_O (3 × 10 mL), vacuum distillation (155 °C, 27 mbar), alcohol **15** (165 mg, 84%) as yellowish oil; R_f_ (hexanes/AcOEt 8:1, 2x) 0.45; ν_max_ (ATR) 3363 (OH), 2916, 2861, 1444, 1375, 1047, 998 cm^−1^; δ_H_ (300 MHz, CDCl_3_) 5.58 (m, 1H, H-6), 5.00 (s, 1H, H-4), 1.77 (m, 3H, Me), 1.69 (s, 3H, H-1), 1.64 (m, 3H, H-7), 1.50, 1.47 (2 × m, 2 × 3H, 2 × Me); δ_C_ (75 MHz, CDCl_3_) 136.3 (C-5), 128.7, 120.0 (C-2, C-3); 117.2 (C-6), 74.3 (C-4), 21.2 (C-1), 20.1, 13.3, (2 × Me), 13.0 (C-7), 11.8 (Me); *m/z* (ESI) 137 (100, M-OH^+^), 138 (11%); HR-MS (HESI): M^+^, found 154.1351. C_10_H_18_O requires 154.1352.

2,3,5,6-Tetramethylhept-2,5-dien-4-ol (**16**) **12** (2.62 mmol, 2.05 equiv), THF (2 mL), −30 °C, ethyl formate (0.12 mL, 1.47 mmol), RT, overnight, NH_4_Cl (10 mL), H_2_O (10 mL), Et_2_O (3 × 15 mL), vacuum distillation (120 °C, 80 mbar), alcohol **16** (175 mg, 71%) as yellowish oil; R_f_ (hexanes/AcOEt 8:1, 2x) 0.43; ν_max_ (ATR) 3339(OH), 2915, 2862, 1446, 1372, 998 cm^−1^; δ_H_ (300 MHz, CDCl_3_) 5.42 (s, 1H, H-4), 1.70, 1.67, 1.65 (2 × m, s, 3 × 6H, H-1, H-7, 4 × Me); δ_C_ (75 MHz, CDCl_3_) 129.8, 127.0 (C-2, C-3, C-5, C-6), 71.1 (C-4), 21.2, 20.0 (C-1, C-7, 2 × Me); 13.7 (2 × Me); *m/z* (ESI) 151 (100, M-OH^+^), 152 (14%); HR-MS (HESI): M^+^, found 168.1508. C_11_H_20_O requires 168.1509.

### 3.4. General experiment for the preparation of (di)enones (**6**–**9**)

To a solution of allyl alcohol in pentane was added activated MnO_2_ (heated at 140 °C/10 Torr for 30 min) at RT under Ar. The suspension was stirred at RT for the indicated time, diluted with diethyl ether, filtered through Celite pad and solids were repeatedly washed with Et_2_O. Filtrate was concentrated in vacuo (34 °C, 550 mbar) to furnish a corresponding enone pure by NMR. For analytical purposes, an aliquot was purified by either FLC on silica gel or bulb-to-bulb vacuum distillation.

2,3-Dimethylnon-2-en-4-one (**6**) Alcohol **13** (200 mg, 1.18 mmol), pentane (4 mL), MnO_2_ (2.05 g, 23.60 mmol, 20 equiv), RT, 4 d, Et_2_O (10 mL), Celite (2 × 1 cm), Et_2_O (4 × 10 mL), vacuum distillation (120 °C, 80 mbar), enone **6** (140 mg, 71%) as a colourless oil; R_f_ (hexanes/AcOEt 10:1) 0.50; ν_max_ (ATR) 2956, 2928, 2860, 1685 (C=O), 1456, 1375, 1043, 1013 cm^−1^; δ_H_ (300 MHz, CDCl_3_) 2.50 (dd, *J* = 7.3 Hz, 2H, H-5), 1.81, 1.73 (m, 6H, s, 3H, H-1, 2 × Me), 1.30 (m, 6H, H-6, H-7, H-8), 0.89 (t, *J* = 6.8 Hz, 3H, H-9); δ_C_ (75 MHz, CDCl_3_) 208.9 (C=O), 136.0, 131.8, (C-2, C-3), 41.9 (C-5), 31.7 (C-6), 23.9 (C-7), 22.7 (C-8), 22.4, 21.3, 15.6, 14.1 (4 × Me); *m/z* (ESI) 169 (100, M + H^+^), 170 (11%); HR-MS (HESI): M^+^, found 168.1508. C_11_H_20_O requires 168.1508.

2,3,5,5-Tetramethylhept-2-en-4-one (**7**) Alcohol **14** (230 mg, 1.35 mmol), pentane (4 mL), MnO_2_ (2.35 g, 27.0 mmol, 20 equiv), RT, 72 h, Et_2_O (10 mL), Celite (2 × 1 cm), Et_2_O (4 × 10 mL), enone **7** (175 mg, 76%) as a colourless oil; R_f_ (hexanes/AcOEt 10:1) 0.53; ν_max_ (ATR) 2967, 2932, 2880, 1682 (C=O), 1462, 1376, 1002, 973 cm^−1^; δ_H_ (300 MHz, CDCl_3_) 1.72, 1.63, 1.56 (3 × m, 3 × 3H, 3 × Me), 1.55 (q, 2H, *J* = 7.4 Hz, H-6), 1.1 (s, 6H, 2 × Me), 0.82 (t, *J* = 7.4 Hz, 3H, H-7); δ_C_ (75 MHz, CDCl_3_) 218.1 (C=O), 131.9, 128.9 (C-2, C-3), 47.7 (C-5), 32.8 (C-6), 24.5 (2 × Me), 22.4, 19.4, 16.2 (C-1, 2 × Me), 8.9 (C-7); *m/z* (ESI) 169 (100, M + H^+^), 170 (12%); HR-MS (HESI): M^+^, found 168.1506. C_11_H_20_O requires 168.1509.

(*E*)-2,3,5-Trimethylhept-2,5-dien-4-one (**8**) Alcohol **15** (113 mg, 0.73 mmol), pentane (3 mL), MnO_2_ (1.28 g, 14.68 mmol, 20 equiv), RT, 48 h, Et_2_O (5 mL), Celite (2 × 1 cm), Et_2_O (3 × 10 mL), enone **8** (89 mg, 74%) as a colourless oil; R_f_ (pentane/Et_2_O 10:1) 0.64; ν_max_ (ATR) 2979, 2918, 2860, 1639 (C=O), 1444, 1376, 1285, 1045 cm^−1^; δ_H_ (300 MHz, CDCl_3_) 6.64 (m, 1H, H-6), 1.86 (m, 3H, H-7), 1.80, 1.75, 1.72, 1.54 (3 × m, s, 4 × 3H, H-1, 3 × Me); δ_C_ (75 MHz, CDCl_3_) 204.3 (C=O), 141.6 (C-6), 137.7, 130.6, 130.0 (C-2, C-3, C-5), 22.2, 19.8, 17.0, 15.1, 10.5 (C-1, C-7, 3 × Me); *m/z* (ESI) 153 (100, M + H^+^), 154 (10%); HR-MS (HESI): M^+^, found 152.1194. C_10_H_16_O requires 152.1196.

(*E*)-2,3,5,6-Tetramethylhept-2,5-dien-4-one (**9**) Alcohol **16** (113 mg, 0.67 mmol), pentane (2 mL), MnO_2_ (1.17 g, 13.45 mmol, 20 equiv), RT, 4 d, Et_2_O (5 mL), Celite (2 × 1 cm), Et_2_O (3 × 10 mL), enone **9** (85 mg, 76%) as a colourless oil; R_f_ (pentane/Et_2_O 10:1) 0.64; ν_max_ (ATR) 2915, 2863, 1629 (C=O), 1445, 1373, 1294, 1003 cm^−1^; δ_H_ (300 MHz, CDCl_3_) 1.82, 1.79, 1.76 (3 × m, 3 × 6H, H-1, H-7, 4 × Me); δ_C_ (75 MHz, CDCl_3_) 204.7 (C=O), 138.1, 132.1 (C-2, C-3, C-5, C-6), 22.0, 21.7 (C-1, C-7, 2 × Me), 15.6 (2 × Me); *m/z* (ESI) 167 (100, M + H^+^), 168 (11%); HR-MS (HESI): M^+^, found 166.1952. C_11_H_18_O requires 166.1952.

2,5-Diisopropylidene-cyclopentanone (**10**) Prepared according to the reported procedure [9c] as a colourless oil; R_f_ (hexanes/AcOEt 10:1) 0.51; ν_max_ (ATR) 2956, 2905, 2849 (C-H), 1682, 1613 (C=O), 1435, 1363, 1267, 1195, 978, 796 cm^−1^; δ_H_ (300 MHz, CDCl_3_) 2.51 (s, 4H, H-3, H-4), 2.27 (s, 6H, 2 × Me), 1.81 (s, 6 H, 2 × Me); δ_C_ (75 MHz, CDCl_3_) 196.6 (C=O), 146.2, 134.4 (4 × C_q_), 25.4 (2 × CH_2_), 24.4, 20.4 (4 × Me); *m/z* (ESI) 165 (100, M + H^+^), 166 (13%); HR-MS (HESI): M^+^, found 164.1195. C_11_H_16_O requires 164.1196.

## 4. Conclusions

We have performed a chiral-pool-based chemoenzymatic synthesis of enantioenriched (*S*)-5-methylhept-2-en-4-one **1** in 39% overall yield and with 73% *ee*. This four-step aldol-based sequence employs natural substrates and innocuous reactions, avoids the use of highly reactive and/or toxic reagents, does not require anhydrous conditions and uses only distillation as a purification method. Thus, such methodology represents a green and scalable alternative to only two approaches towards enantioenriched **1** known so far.

In addition, we have designed and prepared (di)enones **6**–**9** as achiral synthetic analogues of the naturally occurring **1**. Their short two-step preparation features the initial alkenyl lithium addition to carbonyl electrophiles with subsequent allylic oxidation of alkenols to desired targets. The results of their sensory analysis clearly show that relatively minor structural changes of the natural’s molecule significantly alter its olfactory properties. Thus, simple (poly)methylation completely changes the original hazelnut aroma of (*S*)-5-methylhept-2-en-4-one **1** and shifts the odour of its analogues **6**–**9** to eucalyptus, menthol, camphor, and sweet aroma, respectively.

## Figures and Tables

**Table 1:** Olfactory properties of compounds **6**–**10**, **13**–**16** prepared via Scheme 2.

Main Odour(s)	Compound	Minor Aspect(s)	Odour Intensity
Eucalyptus Menthol Camphor	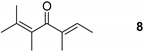	Fresh, spicy, floral	Strong
	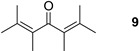	Herbal, medicinal	Strong
	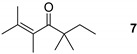	Floral	Mild
	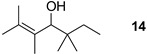	Terpenic	Mild
	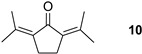	Medicinal, sweet, oily	Mild
Sweet	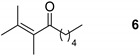	Tropical, fruity, spicy	Strong
Root vegetables Earthy	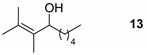	Dried poppy-head	Mild
	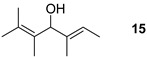	Celery, lovage	Mild
	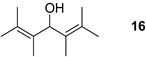	Parsley, celery, sprouting potatoes	Mild

## Supplementary Materials

The following are available online. Copies of spectra (chiral GC, NMR) of prepared compounds are available.
