# Supplementary material for: Scalable Preparation of Enantioenriched (S)-5-methylhept-2-en-4-one. Synthesis and Aroma Properties of Achiral Analogues Thereof
Source: Molecules. 2019 Dec 8;24(24):4497. doi: 10.3390/molecules24244497 (PMC6943495; doi:10.3390/molecules24244497)
Supplement: Supplementary file 1 [file molecules-24-04497-s001.pdf]

SUPPLEMENTARY MATERIAL

Scalable preparation of enantioenriched (*S*)-5-methylhept-2-en-4-one. Synthesis and properties of achiral analogues of thereof

Eva Puchľová[a], Michal Dendys[a], Ivan Špánik[b] and Peter Szolcsányi[a,*]

[a] *Department of Organic Chemistry, Slovak University of Technology, Radlinského 9, SK-812 37 Bratislava, Slovakia*

[b] *Department of Analytical Chemistry, Slovak University of Technology, Radlinského 9, SK-812 37 Bratislava, Slovakia*

CONTENTS:

1. Materials and Methods (pp. 2-3)

2. Synthetic Procedures and Analytical Data (pp. 3-8)

[*] Corresponding author. Tel.: +421-2-593-25745; e-mail: peter.szolcsanyi@stuba.sk

1. MATERIALS AND METHODS

Chemicals and reagents were purchased from commercial sources (Alfa Aesar, Sigma-Aldrich) and were used without further purification. In case of anhydrous solvents these were prepared either by filtration through a column of activated alumina or by standing over activated 4Å molecular sieves and stored under argon atmosphere. Hexanes refer to a mixture of C-6 alkanes (b.p. 60–80°C). Yields refer to chromatographically and spectroscopically (^{1}H NMR) homogeneous material, unless otherwise stated. Reactions were monitored by thin layer chromatography (TLC) carried out on aluminium sheets pre-coated with silica gel 60 F_{254} (Merck) or aluminium oxide 60 F_{254} (neutral, Merck). Visualisation was performed using shortwave UV light followed by dipping TLC plates in either basic solution of $KMnO_4$, acidic solution of vanillin or acidic solution of ceric ammonium nitrate followed by heating with a heat gun. Flash column chromatography (FLC) was performed using Silica Gel 60 (particle size 0.040-0.063 mm). NMR spectra were recorded in $CDCl_3$ on a Varian INOVA 300 (300 MHz for ^{1}H, 75 MHz for ^{13}C nuclei) or Varian VNMRS 600 (600 MHz for ^{1}H, 151 MHz for ^{13}C nuclei) NMR spectrometer and were correctly shifted using residual non-deuterated solvent or tetramethylsilane as an internal reference ($CHCl_3$: δ_H = 7.26 ppm, δ_C = 77.16 ppm (central peak of a 1:1:1 triplet), TMS: $\delta_H = \delta_C$ = 0.00 ppm). Chemical shifts (δ) are quoted in ppm. LC-MS analyses were performed on Agilent 1200 Series instrument equipped with a multimode MS detector using the MM ESI/APCI ionisation method (column Zorbax Eclipse XDB-18, 150 x 4.6 mm, particle size 5 µm, eluent water with 0.1% HCO_2H /CH_3CN, 70:30, flow 1.5 mL/min). GC analyses were performed on a gas chromatograph Agilent 7820A equipped with FID and a split–splitless injector (column DB-5 30 m x 0.25 mm x 0.25 µm, injection 0.1 µL, split 20:1, temperature gradient 40 °C (2 min) → 15 °C/min → 220 °C (15 min), carrier gas H_2, flow 1.2 mL/min). Chiral GC analyses were performed on a gas chromatograph Agilent 7890A equipped with FID and a split–splitless injector (column Cyclosil-B 30 m x 0.32 mm x 0.25 µm, injection 0.2 µL, split 50:1, temperature gradient 40 °C (0 min) → 10 °C/min → 80 °C (0 min) → 25 °C/min → 220 °C (2 min), carrier gas H_2, flow 2.0 mL/min). GC-MS analyses were performed on a gas chromatograph Agilent 7890A and coupled with Agilent 5975C inert MSD with Triple-Axis Detector (column DB-Wax 30 m x 0.25 mm x 0.15 µm, injection 1 µL, split 20:1, temperature gradient 40 °C (2 min) → 15 °C/min → 220 °C (15 min), carrier gas H_2, flow 1.2 mL/min). High-resolution mass spectra (HRMS) were recorded on a Thermo Scientific Orbitrap Velos mass spectrometer with a heated electrospray ionisation (HESI) source in positive and/or negative mode. FTIR spectra

were obtained on a Nicolet 5700 spectrometer (Thermo Electron) equipped with a Smart Orbit (diamond crystal ATR) accessory using the reflectance technique (4000–400 cm^{-1}). The sensory analysis was performed by authors in a clean and odourless environment at 22°C. The prepared compounds were evaluated as 10% solutions in aq. ethanol (95% w/w) by using testing stripes.

2. SYNTHETIC PROCEDURES AND ANALYTICAL DATA

*(S)-Ethyl 4-methyl-3-oxohexanoate (**3**)*

A mixture of potassium ethyl malonate (100.0 g, 0.587 mol, 1.5 equiv) was treated with magnesium chloride (56.0 g, 0.587 mol, 1.5 equiv) in tetrahydrofuran (360 mL), and the resulting grey slurry was stirred at 60 °C for 5 h. During that time, in a separate reaction vessel, a soln. of (*S*)-2-methylbutanoic acid **2** (88% *ee*, 40.0 g, 0.343 mol) in THF (160 mL) was added to a soln. of carbonyldiimidazol (66.0 g, 0.407 mol, 1.2 equiv) in THF (140 mL) and the resulting yellow solution was stirred at 28 °C for 4 h. Then, after 5 h reaction time, a THF soln. of mixture containing malonate and $MgCl_2$ was cooled to r.t. and a THF soln. of crude acyl imidazole formed from acid **2** was added dropwise over 30 min. The resulting white suspension was stirred at 50 °C for 5 h and subsequently at r.t. overnight. The reaction mixture was then added to 1M aq. HCl soln. (1600 mL). The resulting pale-yellow solution was stirred at r.t. for 30 min, ethyl acetate (600 mL) was added, phases were separated, and aq. layer was extracted with EtOAc (600 mL). Combined org. extracts were sequentially washed with 1M aq. HCl soln. (400 mL), water (500 mL), 2% aq. Na_2CO_3 soln. (700 mL), water (500 mL), and brine (500 mL), subsequently dried over anhydr. Na_2SO_4 and concentrated *in vacuo* to give pale-yellow oil (76.23 g). The crude product was purified by vacuum distillation (b.p. 64-67 °C/3.4 mbar) to yield (*S*)-ketoester **3** (60.54 g, 90%) as a colourless liquid; ^{1}H and ^{13}C NMR spectra of (*S*)-**3** are in full accordance with literature data[1] for racemic **3**; in addition, signals of the enol form of **3** are clearly detectable in its proton spectrum measured in deuterochloroform: δ_H (400 MHz, $CDCl_3$) 12.11 (d, *J* = 0.7 Hz, 1 H), 4.97–4.96 (m, 1 H), 4.18 (q, *J* = 7.1 Hz, 2 H), 2.15 (m, *J* = 7.0 Hz, 1 H), 1.79–1.62 (m, 1 H), 1.42 (m, 1 H), 1.29 (t, *J* = 7.1 Hz, 3 H), 1.12 (d, *J* = 6.9 Hz, 3 H), 0.89 (t, *J* = 7.4 Hz, 3 H);

[1] a) Appel, W. P.; Portale, G.; Wisse, E.; Dankers, P. Y.; Meijer, E. W. *Macromolecules* **2011**, *44*, 6776–6784; b) Crombie, L.; Jones, R. C.; Palmer, C. J. *J. Chem. Soc., Perkin Trans. 1* **1987**, 317–331; c) Schweizer, E.; Gaich, T.; Brecker, L.; Mulzer, J. *Synthesis* **2007**, *24*, 3807–3814.

GC: t_R = 7.73 min (keto-form), t_R = 7.54 min (enol-form); GC-MS: *m/z* (%) 172 (6, M^+), 157 (1), 144 (9), 127 (3), 115 (27), 98 (5), 85 (31), 69 (12), 57 (100), 43 (36).

(S)-4-Methyl-3-oxohexanoic acid (4)

To a soln. of ketoester **3** (8.0 g, 46.5 mmol) in an aq. sodium phosphate buffer (32 mL, pH ~ 7) was added Novozym 435 (400 mg) and the suspension was stirred at r.t. for 22 h, while the pH was kept neutral by addition of aq. NaOH. Solids were filtered off and the filtrate containing ketoacid **4** (100% GC yield, 88% *ee*) was directly used for the subsequent Knoevenagel condensation with acetaldehyde. The enantiomeric purity of crude **4** was determined by chiral GC *via* (*S*)-3-methylpent-2-on (t_R = 3.53 min) formed *in situ* by thermal decarboxylation of **4** during analysis.

(S)-2-Hydroxy-5-methylhept-4-one (5)

To a soln. of crude ketoacid **4** (8 g, 46.5 mmol) in an aq. phosphate buffer (42.5 mL) was added tetrabutylammonium hydrogen sulphate (79 mg) and the pH was adjusted to *ca.* 8 by aq. NaOH soln. Acetaldehyde (2.6 mL, 51.4 mmol, 1.1 equiv) was added and the resulting soln. was stirred at r.t. for 1.5 h and then at 40 °C for 21 h. The reaction mixture was extracted with diethyl ether (3 x 70 mL), separated org. layer was dried over anhydr. Na_2SO_4 and the solvent was evaporated *in vacuo* to yield pale-yellow liquid (6.865 g). The crude product was purified by vacuum distillation (b.p. 63-64 °C/3.6 mbar) to afford ketol **5** (3.577 g, 53%) as a colourless liquid; δ_H (600 MHz, $CDCl_3$) 4.23–4.17 (m, 1H, H-2), 3.01 (bs, 1H, exchange with D_2O, OH), 2.62 (ddd, *J* = 17.8, 13.0, 2.8 Hz, 1H, H-3a), 2.51 (ddd, *J* = 17.8, 13.5, 9.0 Hz, 1H, H-3b), 2.46–2.39 (m, 1H, H-5), 1.72–1.63 (m, 1H, H-6a), 1.43–1.35 (m, 1H, H-6b), 1.18 (dd, *J* = 6.4, 0.7 Hz, 3H, H-1), 1.06 (dd, *J* = 7.0, 1.3 Hz, 3H, Me), 0.87 (t, *J* = 7.5 Hz, 3H, H-7), NMR spectrum is in accordance with the literature data[2]; GC: t_R = 7.67 min; GC-MS: *m/z* (%) 144 (2, M^+), 116 (4), 103 (18), 87 (85), 85 (26), 69 (23), 57 (75), 43 (100).

(E,S)-5-Methylhept-2-en-4-one (1)

To a mixture of ketol **5** (2.85 g, 19.8 mmol) in cyclohexane (49 mL) was added *p*-toluenesulfonic acid monohydrate, (190 mg, 1.0 mmol, 0.05 equiv) and resulting mixture was stirred at 70 °C for 2.5 h. Subsequently, the mixture was washed with sat. aq. $NaHCO_3$ soln. (25 mL), aq. phase was extracted with diethyl ether (3 x 60 mL), org. phase was dried over

[2] Seebach, D.; Ehrig, V.; Teschner, M. *Justus Liebigs Ann. Chem.* **1976**, 7/8, 1357–1369.

anhydr. Na_2SO_4 and concentrated *in vacuo* to give colourless liquid (2.60 g). The crude product was purified by bulb-to-bulb vacuum distillation (b.p. 75-76 °C/4 mbar) to afford a (*S*)-enantioenriched enone **1** (2.03 g, 82% yield, 73% *ee*) as a colourless liquid; NMR spectra of (*S*)-**1** are in full accordance with the literature data[3]; Chiral GC: t_R = 5.65 min; GC-MS: *m/z* (%) 126 (1, M^+), 111 (11), 98 (12), 69 (100), 57 (4), 41 (21).

General experiment for the preparation of (di)allyl alcohols (13)–(16)

To a freshly prepared soln. of 1,2-dimethylpropenyl lithium **12** in anhydr. THF was added the respective aldehyde or ester dropwise at low temperature over 5 min. under Ar. The mixture was gradually warmed to rt and stirred overnight. The reaction was quenched with sat. aq. NH_4Cl soln., diluted with water and extracted with diethyl ether. Combined org. extracts were dried over $MgSO_4$ and concentrated *in vacuo* (34 °C, 650 → 250 mbar). Crude product was purified by bulb-to-bulb vacuum distillation to furnish a corresponding allyl alcohol **13**–**16**.

*2,3-Dimethylnon-2-en-4-ol **(13)***

12 (1.35 mmol, 1.05 equiv), THF (2 mL), -50 °C, hexanal (0.16 mL, 1.28 mmol), rt, overnight, NH_4Cl (5 mL), H_2O (5 mL), Et_2O (3 x 10 mL), vacuum distillation (130 °C, 50 mbar), alcohol **13** (165 mg, 84%) as colourless oil; R_f (hexanes/AcOEt 4:1) 0.43; ν_{max} (ATR) 3342 (OH), 2955, 2927, 2859, 1457, 1375, 1010 cm^{-1}; δ_H (300 MHz, $CDCl_3$) 4.63 (t, *J* = 6.96 Hz, 1H, H-4), 1.71, 1.67, 1.61 (s, 2 x m, 3 x 3H, H-1, 2 x Me), 1.40 (m, 8H, H-5, H-6, H-7, H-8), 0.89 (m, 3H, H-9); δ_C (75 MHz, $CDCl_3$) 129.6, 127.4 (C-2, C-3), 71.2 (C-4), 35.1 (C-5), 31.9 (C-7), 25.6 (C-6), 22.7 (C-8), 21.1, 19.8 (C-1, Me); 14.1 (C-9), 11.6 (Me); *m/z* (ESI) 153 (100, M-OH^+), 154 (14%); HRMS (HESI): M^+, found 170.1665. $C_{11}H_{22}O$ requires 170.1665.

*2,3,5,5-Tetramethylhept-2-en-4-ol **(14)***

12 (2.62 mmol, 1.05 equiv), THF (2 mL), -30 °C, 2,2-dimethylbutanal (0.31 mL, 2.5 mmol), rt, overnight, NH_4Cl (10 mL), H_2O (10 mL), Et_2O (3 x 15 mL), vacuum distillation (130 °C, 80 mbar), alcohol **14** (285 mg, 67%) as yellowish oil; R_f (hexanes/AcOEt 4:1) 0.59; ν_{max} (ATR) 3398 (OH), 2962, 2917, 2879, 1462, 1373, 1002 cm^{-1}; δ_H (300 MHz, $CDCl_3$) 4.42

[3] a) Jauch, J.; Schmalzing, D.; Schurig, V.; Emberger, R.; Hopp, R.; Köpsel, M.; Silberzahn, W.; Werkhoff, P. *Angew. Chem., Int. Ed.* **1989**, 28, 1022–1023; b) Jauch, J.; Czesla, H.; Schurig, V. *Tetrahedron* **1999**, 55, 9787–9792.

(d, J = 3.75 Hz, 1H, H-4), 1.69, 1.66 (2 x m, 3H, 6H, H-1, 2 x Me), 1.35 (d, J = 3.75 Hz, OH), 1.31 (m, 2H, H-6), 0.86 (m, 3H, H-7), 0.88, 0.80 (2 x s,2 x 3H, 2 x Me); δ_C (75 MHz, $CDCl_3$) 128.4, 129.2 (C-2, C-3), 76.9 (C-4), 39.7 (C-5), 31.9 (C-6), 23.5, 22.6 (2 x Me), 21.4, 21.3 (C-1, Me); 14.6 (Me), 8.5 (C-7); *m/z* (ESI) 153 (100, M-OH^+), 154 (12%); HRMS (HESI): M^+, found 170.1665. $C_{11}H_{22}O$ requires 170.1665.

*(E)-2,3,5-Trimethylhept-2,5-dien-4-ol (**15**)*

12 (1.35 mmol, 1.05 equiv), THF (2 mL), -45 °C, (*E*)-2-methylbutanal (0.13 mL, 1.28 mmol), rt, overnight, NH_4Cl (5 mL), H_2O (5 mL), Et_2O (3 x 10 mL), vacuum distillation (155 °C, 27 mbar), alcohol **15** (165 mg, 84%) as yellowish oil; R_f (hexanes/AcOEt 8:1, 2x) 0.45; ν_{max} (ATR) 3363 (OH), 2916, 2861, 1444, 1375, 1047, 998 cm^{-1}; δ_H (300 MHz, $CDCl_3$) 5.58 (m, 1H, H-6), 5.00 (s, 1H, H-4), 1.77 (m, 3H, Me), 1.69 (s, 3H, H-1), 1.64 (m, 3H, H-7), 1.50, 1.47 (2 x m, 2 x 3H, 2 x Me); δ_C (75 MHz, $CDCl_3$) 136.3 (C-5), 128.7, 120.0 (C-2, C-3); 117.2 (C-6), 74.3 (C-4), 21.2 (C-1), 20.1, 13.3, (2 x Me), 13.0 (C-7), 11.8 (Me); *m/z* (ESI) 137 (100, M-OH^+), 138 (11%); HRMS (HESI): M^+, found 154.1351. $C_{10}H_{18}O$ requires 154.1352.

*2,3,5,6-Tetramethylhept-2,5-dien-4-ol (**16**)*

12 (2.62 mmol, 2.05 equiv), THF (2 mL), -30 °C, ethyl formate (0.12 mL, 1.47 mmol), rt, overnight, NH_4Cl (10 mL), H_2O (10 mL), Et_2O (3 x 15 mL), vacuum distillation (120 °C, 80 mbar), alcohol **16** (175 mg, 71%) as yellowish oil; R_f (hexanes/AcOEt 8:1, 2x) 0.43; ν_{max} (ATR) 3339 (OH), 2915, 2862, 1446, 1372, 998 cm^{-1}; δ_H (300 MHz, $CDCl_3$) 5.42 (s, 1H, H-4), 1.70, 1.67, 1.65 (2 x m, s, 3 x 6H, H-1, H-7, 4 x Me); δ_C (75 MHz, $CDCl_3$) 129.8, 127.0 (C-2, C-3, C-5, C-6), 71.1 (C-4), 21.2, 20.0 (C-1, C-7, 2 x Me); 13.7 (2 x Me); *m/z* (ESI) 151 (100, M-OH^+), 152 (14%); HRMS (HESI): M^+, found 168.1508. $C_{11}H_{20}O$ requires 168.1509.

General experiment for the preparation of (di)enones (6)–(9)

To a soln. of allyl alcohol in pentane was added activated MnO_2 (heated at 140 °C/10 Torr for 30 min) at rt under Ar. The suspension was stirred at rt for the indicated time, diluted with diethyl ether, filtered through Celite pad and solids were repeatedly washed with Et_2O. Filtrate was concentrated *in vacuo* (34 °C, 550 mbar) to furnish a corresponding enone pure by NMR. For analytical purposes, an aliquot was purified by either FLC on silica gel or bulb-to-bulb vacuum distillation.

*2,3-Dimethylnon-2-en-4-one (**6**)*

Alcohol **13** (200 mg, 1.18 mmol), pentane (4 mL), MnO_2 (2.05 g, 23.60 mmol, 20 equiv), rt, 4 d, Et_2O (10 mL), Celite (2 x 1 cm), Et_2O (4 x 10 mL), vacuum distillation (120 °C, 80 mbar), enone **6** (140 mg, 71%) as a colourless oil; R_f (hexanes/AcOEt 10:1) 0.50; ν_{max} (ATR) 2956, 2928, 2860, 1685 (C=O), 1456, 1375, 1043, 1013 cm^{-1}; δ_H (300 MHz, $CDCl_3$) 2.50 (dd, J = 7.3 Hz, 2H, H-5), 1.81, 1.73 (m, 6H, s, 3H, H-1, 2 x Me), 1.30 (m, 6H, H-6, H-7, H-8), 0.89 (t, J = 6.8 Hz, 3H, H-9); δ_C (75 MHz, $CDCl_3$) 208.9 (C=O), 136.0, 131.8, (C-2, C-3), 41.9 (C-5), 31.7 (C-6), 23.9 (C-7), 22.7 (C-8), 22.4, 21.3, 15.6, 14.1 (4 x Me); *m/z* (ESI) 169 (100, $M+H^+$), 170 (11%); HRMS (HESI): M^+, found 168.1508. $C_{11}H_{20}O$ requires 168.1508.

2,3,5,5-Tetramethylhept-2-en-4-one (7)

Alcohol **14** (230 mg, 1.35 mmol), pentane (4 mL), MnO_2 (2.35 g, 27.0 mmol, 20 equiv), rt, 72 h, Et_2O (10 mL), Celite (2 x 1 cm), Et_2O (4 x 10 mL), enone **7** (175 mg, 76%) as a colourless oil; R_f (hexanes/AcOEt 10:1) 0.53; ν_{max} (ATR) 2967, 2932, 2880, 1682 (C=O), 1462, 1376, 1002, 973 cm^{-1}; δ_H (300 MHz, $CDCl_3$) 1.72, 1.63, 1.56 (3 x m, 3 x 3H, 3 x Me), 1.55 (q, 2H, J = 7.4 Hz, H-6), 1.1 (s, 6H, 2 x Me), 0.82 (t, J = 7.4 Hz, 3H, H-7); δ_C (75 MHz, $CDCl_3$) 218.1 (C=O), 131.9, 128.9 (C-2, C-3), 47.7 (C-5), 32.8 (C-6), 24.5 (2 x Me), 22.4, 19.4, 16.2 (C-1, 2 x Me), 8.9 (C-7); *m/z* (ESI) 169 (100, $M+H^+$), 170 (12%); HRMS (HESI): M^+, found 168.1506. $C_{11}H_{20}O$ requires 168.1509.

*(E)-2,3,5-Trimethylhept-2,5-dien-4-one (**8**)*

Alcohol **15** (113 mg, 0.73 mmol), pentane (3 mL), MnO_2 (1.28 g, 14.68 mmol, 20 equiv), rt, 48 h, Et_2O (5 mL), Celite (2 x 1 cm), Et_2O (3 x 10 mL), enone **8** (89 mg, 74%) as a colourless oil; R_f (pentane/Et_2O 10:1) 0.64; ν_{max} (ATR) 2979, 2918, 2860, 1639 (C=O), 1444, 1376, 1285, 1045 cm^{-1}; δ_H (300 MHz, $CDCl_3$) 6.64 (m, 1H, H-6), 1.86 (m, 3H, H-7), 1.80, 1.75, 1.72, 1.54 (3 x m, s, 4 x 3H, H-1, 3 x Me); δ_C (75 MHz, $CDCl_3$) 204.3 (C=O), 141.6 (C-6), 137.7, 130.6, 130.0 (C-2, C-3, C-5), 22.2, 19.8, 17.0, 15.1, 10.5 (C-1, C-7, 3 x Me); *m/z* (ESI) 153 (100, $M+H^+$), 154 (10%); HRMS (HESI): M^+, found 152.1194. $C_{10}H_{16}O$ requires 152.1196.

*(E)- 2,3,5,6-Tetramethylhept-2,5-dien-4-one (**9**)*

Alcohol **16** (113 mg, 0.67 mmol), pentane (2 mL), MnO_2 (1.17 g, 13.45 mmol, 20 equiv), rt, 4 d, Et_2O (5 mL), Celite (2 x 1 cm), Et_2O (3 x 10 mL), enone **9** (85 mg, 76%) as a

colourless oil; R_f (pentane/Et_2O 10:1) 0.64; ν_{max} (ATR) 2915, 2863, 1629 (C=O), 1445, 1373, 1294, 1003 cm^{-1}; δ_H (300 MHz, $CDCl_3$) 1.82, 1.79, 1.76 (3 x m, 3 x 6H, H-1, H-7, 4 x Me); δ_C (75 MHz, $CDCl_3$) 204.7 (C=O), 138.1, 132.1 (C-2, C-3, C-5, C-6), 22.0, 21.7 (C-1, C-7, 2 x Me), 15.6 (2 x Me); *m/z* (ESI) 167 (100, $M+H^+$), 168 (11%); HRMS (HESI): M^+, found 166.1952. $C_{11}H_{18}O$ requires 166.1952.
